# Possibilities, Problems, and Perspectives of Data Collection by Mobile Apps in Longitudinal Epidemiological Studies: Scoping Review

**DOI:** 10.2196/17691

**Published:** 2021-01-22

**Authors:** Florian Fischer, Sina Kleen

**Affiliations:** 1 Institut of Public Health Charité - Universitätsmedizin Berlin Berlin Germany; 2 Institute of Gerontological Health Services and Nursing Research Ravensburg-Weingarten University of Applied Sciences Weingarten Germany; 3 Department of Population Medicine and Health Services Research School of Public Health Bielefeld University Bielefeld Germany

**Keywords:** apps, questionnaire, survey, epidemiology, healthcare

## Abstract

**Background:**

The broad availability of smartphones and the number of health apps in app stores have risen in recent years. Health apps have benefits for individuals (eg, the ability to monitor one’s health) as well as for researchers (eg, the ability to collect data in population-based, clinical, and observational studies). Although the number of health apps on the global app market is huge and the associated potential seems to be great, app-based questionnaires for collecting patient-related data have not played an important role in epidemiological studies so far.

**Objective:**

This study aims to provide an overview of studies that have collected patient data using an app-based approach, with a particular focus on longitudinal studies. This literature review describes the current extent to which smartphones have been used for collecting (patient) data for research purposes, and the potential benefits and challenges associated with this approach.

**Methods:**

We conducted a scoping review of studies that used data collection via apps. PubMed was used to identify studies describing the use of smartphone app questionnaires for collecting data over time. Overall, 17 articles were included in the summary.

**Results:**

Based on the results of this scoping review, there are only a few studies that integrate smartphone apps into data-collection approaches. Studies dealing with the collection of health-related data via smartphone apps have mainly been developed with regard to psychosomatic, neurodegenerative, respiratory, and cardiovascular diseases, as well as malign neoplasm. Among the identified studies, the duration of data collection ranged from 4 weeks to 12 months, and the participants’ mean ages ranged from 7 to 69 years. Potential can be seen for real-time information transfer, fast data synchronization (which saves time and increases effectivity), and the possibility of tracking responses longitudinally. Furthermore, smartphone-based data-collection techniques might prevent biases, such as reminder bias or mistakes occurring during manual data transfers. In chronic diseases, real-time communication with physicians and early detection of symptoms enables rapid modifications in disease management.

**Conclusions:**

The results indicate that using mobile technologies can help to overcome challenges linked with data collection in epidemiological research. However, further feasibility studies need to be conducted in the near future to test the applicability and acceptance of these mobile apps for epidemiological research in various subpopulations.

## Introduction

Worldwide, there were 2.39 billion smartphone users in 2017, and this number is predicted to exceed three billion by 2021 [1]. Undoubtedly, smartphones have become part of everyday life for many people around the world. Alongside the increase in smartphone use, the market for smartphone apps has risen massively in recent years [2]. In health care, it is anticipated that apps have the potential to decrease both spatial and administrative barriers [2]. However, removing barriers is only one potential outcome resulting from the use of these applications. The market for mHealth apps is growing rapidly. In 2015 alone, there were more than 100,000 new health apps published in relevant app stores, with approximately three billion downloads of apps related to health, fitness, and medicine [2]. In comparison to 2013, the number of downloaded apps has almost doubled [2]. Consumers use apps to track steps, heart rate, sleep patterns, and so forth. Smartphones also function as new tools for measuring the health of individuals. Moreover, smartphone apps may deliver data at the population level. For that reason, smartphones are expected to be beneficial to health care research and epidemiological studies. Over the last two decades, surveys have ceased to exclusively use traditional methods of data collection, such as paper or telephone-based questionnaires; questionnaires have instead been developed using electronic systems, such as internet-based surveys and personal digital assistants. Although there has been a huge rise in the use of smartphones, issues regarding the benefits and potential uses of app-based questionnaires still need to be addressed [3]. Various studies have developed smartphone apps for educational or communication purposes for medical school students and clinicians [4]. However, only a few questionnaires on smartphone apps have been used in clinical settings, such as in sleep disorder tracking and for the administration of psychiatric questionnaires [5-7]. Beyond epidemiology, public health, and health services research, there are approaches in the field of cognitive science that have also detected the potential for data collection via smartphone apps [8].

Although data collection using smartphone apps has not yet been comprehensively studied and is not very frequently used, the availability of and access to smartphones among the world’s population raises the potential for large-scale surveys in (longitudinal) population-based studies. This scoping review focuses on studies investigating the acceptability, feasibility, and performance of mobile apps for data collection in longitudinal studies, irrespective of the studies’ purposes (eg, measuring vital parameters, providing questionnaires) or the types of apps in question (eg, apps that are medical products prescribed by physicians or those that are lifestyle products selected by users). To guide the scoping review, we focused on the following 4 overarching research questions: 1) which types of studies (feasibility/pilot vs full-scale) have been conducted to date, 2) over what period of time has data been collected within these studies, 3) which specific target groups (eg, children, elderly, migrants) have been included in the studies, and 4) what potential benefits and challenges to app-based data collection are described in the studies?

## Methods

We conducted a scoping review using PubMed. In the first step, we employed a broad search strategy to include all articles dealing with apps, smartphone devices, or mHealth in the context of data-collection activities. Hence, the following search algorithm was used:

(app[Title/Abstract] OR apps[Title/Abstract] OR smartphone*[Title/Abstract] OR mHealth[Title/Abstract] OR “mobile health”[Title/Abstract] OR mobile phone*[Title/Abstract]) AND (cohort*[Title/Abstract] OR survey*[Title/Abstract] OR questionnaire*[Title/Abstract])

All articles published up to December 31, 2017 were considered by two independent reviewers. This led to 1922 matches after incorporating the predefined filters for studies conducted among humans and those written in the requisite publication language(s) (English or German). The following main exclusion criteria were defined a priori:

- SMS-based approaches (including SMS reminders and supportive text messages in interventional studies)
- Web-based approaches/questionnaires, which can also be conducted on smartphones
- Nomophobia (fear of being out of cellular phone contact)
- Distraction by smartphones while driving
- “App” as an abbreviation for unrelated issues (eg, amyloid precursor protein)
- Screen media time usage among children or adolescents
- Mobile phone access and usage in various populations
- Validation of paper-based compared with software/app-based questionnaires
- Articles dealing with Electronic Health Records
- mHealth interventions using apps only for treatment or educational purposes (eg, limited to disease treatments)

After screening all article titles and abstracts against the criteria listed above, 1808 articles were excluded. This led to 114 articles for which a review of the full text was performed. Due to the large number of articles still remaining, further exclusion criteria were defined and the particular reason for exclusion was documented. Using this procedure, 97 further articles were excluded for the following reasons:

- Cross-sectional study design [9-15]
- Qualitative study design [16]
- Study protocol [17]
- App without purpose of data collection [18-26]
- App only for self-monitoring or willingness to self-monitor disease or certain lifestyle measures (no data storage for researcher) [27-48]
- Mobile-type programs/programmed phones, personal monitors, or ecological momentary assessment protocols displaying on mobile phone screens (without the involvement of an app) [49-52]
- Advanced data-collection systems without the need for data entry by the patient/interviewee (eg, using Global Positioning System location and phone usage data automatically) [53-55]
- App as assistive working tool for researchers or medical staff (no data entry from interviewees/patients) [56-73]
- Study duration less than one month or one-time data entry [47,74-100]
- No possibilities, problems, or perspectives mentioned in the study [101]
- Reviews dealing with smartphone apps, mobile phone surveys, and new data-collection methods in general [102-105]

Thus, studies included in the review met the following criteria:

- Data collection with a smartphone app, including apps used for disease screening
- Apps either prescribed by a physician or selected by the user
- More than self-management functions
- App available on smartphone
- Data entry completed by the interviewees/patients (or close relatives)
- Repeated data entry on a longitudinal basis (more than one month)
- Possibilities, problems, and perspectives of data-collection apps described
- Feasibility studies with attitudes toward
  sharing
  health information (with researchers or physicians) within smartphone apps or smartphone devices

**Figure 1:**
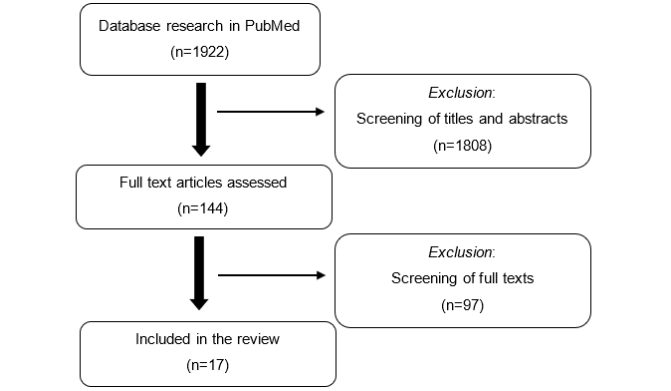
Flowchart of the scoping review.

The final summary consisted of 17 studies, which were summarized using content analysis (Figure 1). There were 3 guiding questions for the content analysis: (1) in which settings have apps for data collection been used so far, (2) what challenges and requirements exist regarding the implementation of apps, and (3) what potential does data collection with smartphone devices have? These aspects are synthesized in the Results based on those aspects described in the primary articles.

## Results

### Overview: Studies Collecting Patient Health Data Over Time

A total of 17 studies were included in the synthesis of the scoping review (Table 1). These studies were conducted for the following reasons:

- To track real-time changes in symptom(s) (severity) or other disease-related patterns (for creating modified/redefined treatment plans) [106-115]
- To determine users’ compliance and engagement with technology adoption, patient data collection, and provider communication with smartphone app [107,111,112,114,115]
- To test an app for aftercare assessment [116-118]
- To screen for certain (disease or patient-related) outcomes [119]
- To develop an app-based participatory surveillance system for collecting syndromic data [120]
- To provide a practical guide to developing and implementing a longitudinal study with an app [121]

Of these 17 studies, 7 were feasibility or pilot studies. The country with the largest number of studies was the US (7/17); 2 studies based in Sweden and 2 more were based in the Netherlands. All (feasibility/pilot and full-scale) studies on data collection over a longer period of time using smartphone devices used a medical or health-related indication, with a wide range of indication areas. The two main indication areas were psychosomatic disorders (eg, depression, mood, post-traumatic stress disorder) and neurodegenerative diseases (eg, Parkinson Disease). The other studies focused on indication areas such as respiratory diseases (eg, asthma), cardiovascular diseases (eg, acute stroke, atrial fibrillation), malignant neoplasms (eg, prostate cancer), sleeping disorders (eg, insomnia, sleep apnea), infectious diseases (eg, acute febrile illness and gastroenteritis), weight loss in obese people, gestational diabetes mellitus, abdominal surgery, chronic pain, and drinking behavior in adolescents. Target groups were related to pregnancy (breastfeeding [108], depression [109], and gestational diabetes mellitus [122]) or focused on children [120] and young people [119].

**Table 1:** Overview of studies included in the scoping review.

Reference	Feasibility or pilot study	Country	Indication area	Study design	Duration of data collection	Participants at baseline, n	Specific target group
Bot et al (2016) [106]	✓	US	Parkinson disease	Observational	6 months	8320	N/A^a^
Burke et al (2017) [121]	✓	US	Obesity	Observational	12 months	151	N/A
Chan et al (2017) [107]	X^b^	US	Asthma	Observational	6 months	7593	N/A
Cooray et al (2015) [116]	X	Sweden	Stroke	Cohort	2 months	48	N/A
Demirci and Bogen (2017) [108]	X	US	Breastfeeding	Observational	8 weeks	61	Pregnancy
Faherty et al (2017) [109]	X	US	Depression	Cohort	8 weeks	36	Pregnancy
Horsch et al (2017) [110]	X	Netherlands	Insomnia	RCT^c^	6-7 weeks (with 3-month follow-up)	151	N/A
Isetta et al (2017) [111]	✓	Spain	Obstructive sleep apnea	Observational	6 weeks	60	N/A
Jamison et al (2017) [112]	✓	US	Chronic pain	Observational	3 months (with option for 6 months)	105	N/A
Labhart et al (2017) [119]	X	Switzerland	Alcohol consumption	Observational	7 weeks	176	Young people
Noe et al (2017) [113]	X	Wales	Mood tracking	Observational	8 weeks	76	N/A
Olson et al (2017) [120]	X	Guatemala	Acute febrile illness and acute gastroenteritis	Cohort	9 months	469	Children
Pavliscsak et al (2016) [117]	✓	England	PTSD and/or TBI in rehabilitation	RCT	36 weeks	95	N/A
Peleg et al (2017) [122]	✓	Italy and Spain	Atrial fibrillation and gestational diabetes mellitus	Cohort	9 months	29	Pregnancy
Silva de Lima et al (2017) [114]	X	Netherlands and North America	Parkinson disease	Observational	6-13 weeks	953	N/A
Sundberg et al (2017) [115]	X	Sweden	Prostate cancer	Intervention	11 weeks	130	N/A
Symer et al (2017) [118]	✓	US	Abdominal surgery	Observational	4 weeks	31	N/A

^a^N/A: not applicable.

^b^Studies marked with an “X” are not feasibility or pilot studies.

^c^RCT: randomized controlled trial.

The sample sizes differed greatly, ranging from 29 to 8320 study participants at baseline. The participants’ mean ages ranged from 7 years [120] (although children did not enter data unsupervised, and so parents were necessarily involved) to 69 years [115], although not every study provided information on the (mean) age of study participants. The majority of studies (10/17) had an observational design, with one using an ecological momentary assessment [121]. Of the observational studies, 4 were cohort studies, 2 were randomized controlled trials, and 1 was a nonrandomized controlled intervention trial. The duration of data collection varied between the studies from 4 weeks to 12 months. Further details are provided in Multimedia Appendix 1.

### Potential of Using Smartphone Devices for Data Collection Over Time

Within the reviewed articles, several potential benefits of using smartphone devices for data collection are described. Firstly, automatic push notifications offer the potential to provide daily, weekly, or monthly reminders or any kind of information [117]. This can be linked to multiple further functions, showing advantages for researchers as well as for users. For that reason, an app can be used as a data-collection tool or as a reminder for the elderly, has potential for self-interventions (eg, when users can track their symptoms), and can be used for real-time information transfer. Patients tracking their symptoms, for example, can use apps to help themselves monitor their diseases, as well as help physicians to create tailor-made treatment plans [117]. Some other advantages can be derived from sharing real-time data between researchers via (secure) platforms as well as storing and replacing data from one software function to another without data loss or mistakes in data entry [106,116]. Rapid data synchronization between data entry (from the user) and demand-oriented provision of the data (for the researcher or the physician) is also possible [116]. Data entry occurs almost simultaneously with its storage and access, which saves time and increases effectivity. With the support of smartphone devices, data collection is possible on a daily basis over long periods of time, and questionnaire responses can be tracked longitudinally [117]. Smartphone-based interventions, or aftercare assessment via the smartphone (in lieu of going to a hospital or seeing a physician), remove barriers and save time; they also remove administrative barriers and travel difficulties (eg, for aftercare assessment). Furthermore, data loss due to reminder bias can be prevented [116].

With sensitive (health) data, there is always a need for data protection. When using secure data storage and unique random codes for user identification, anonymity and data security can be ensured by using apps [106,117].

### Challenges in Using Smartphone Devices for Data Collection Over Time

Although data collection with smartphone apps has great potential, there are also challenges and disadvantages. As is the case for traditional methods of data collection, follow-up with smartphones can be nonuniform [106]. For that reason, users’ (or patients’) engagement and compliance over longer periods of time is a critical issue. Optimally, for conducting studies on smartphone devices, people need to own smartphones with an available mobile internet connection. Otherwise, it is necessary to provide these devices to the participants. This might be feasible for a cross-sectional study of short duration, but for large sample sizes and over longer periods of time this is an obstacle [110]. Furthermore, participant-related preferences are to carry no more than one device [109,121]. Overall, a risk of loss to follow-up exists [108], as in other longitudinal data collections, which might be due to either lack of motivation or technical issues, such as poor data signal or participant discomfort using mobile phones [120]. Therefore, interactive and feedback elements and rewards may lead to greater adherence [112,113].

## Discussion

### Overview

Although the studies identified in this scoping review claimed significant potential for health-related data collection using smartphone devices, it is obvious that very few approaches have integrated these devices into clinical or epidemiological research activities so far. This is particularly true for the data collection in longitudinal study designs. Although apps can be simply tailored to the needs of target groups [12], only a few studies [108,109,120,122] focused explicitly on specified groups. Most of the articles identified in this review dealt with a particular medical or health-related issue. However, apart from pregnancy and childhood, the study participants consisted of nonspecific target groups. For example, we were not able to identify any study particularly focusing on migrant populations. Therefore, the main result of this overview about mobile apps for data collection in epidemiological research is that further studies and, depending on the results of such studies, adequate solutions for implementing modern technologies for data collection in a priori defined target groups is urgently needed. This is also a requirement for achieving high usability. Although it is possible to design an app using free or low-cost development tools, this can be difficult when app designers are unfamiliar with these tools. In such cases, a pilot test with a subpopulation is highly recommended [11]. This is particularly important because a previous study [10] has indicated greater commitment and engagement in using app-based data collection tools, especially among young and middle-aged populations as compared to older populations.

General population surveys frequently do not adequately represent population subgroups. This challenge is reinforced by the fact that specific groups, such as migrants or refugees, are difficult-to-sample populations. These challenges apply to the fact that such difficult-to-sample populations can be rare, difficult to locate, difficult to enumerate, and/or difficult to interview [123]. Therefore, the implementation of mobile apps for data collection might be particularly suitable for vulnerable but also highly mobile populations, such as migrants, in general, or refugees in particular. Accurate data about health status, health determinants, use of health-care services, and risk behaviors among migrants are needed in order to monitor health and improve health services among this population subgroup [124]. As of yet, health-related data on migrants, and refugees in particular, are scarce, and data-collection approaches face several methodological limitations.

As already highlighted in a narrative review of data-collection practices for monitoring migrant health in Europe [124], there are several legal obstacles related to data protection which need to be taken into account. Due to the recently enforced European General Data Protection Regulation [125], aspects of informed consent, privacy and confidentiality, and the right to withdrawal or omit items will receive further attention in the future. These new data protection regulations need to be kept in mind when developing and implementing data-collection tools. These tools should provide additional information to already established instruments such as the census, death registers, and disease surveillance systems. These established instruments only partially include information on migration status, and if they do, the information is only available at a highly aggregated level [126]. Further information on the health of migrants is available based on epidemiological studies of population samples using medical diagnoses [127]. However, these analyses face the challenge that migrants may encounter barriers in accessing health services. This may lead to an underestimation of the disease burden [127].

To overcome these challenges, which lead to an underrepresentation of specific subgroups and, therefore, insufficient data for action [128], several measures have already been taken. These measures include, but are not limited to, disproportionately allocated sampling, multiplicity sampling, and the use of multiple frames. However, it has to be noted that oversampling of people with a migration background is not sufficient to avoid systematic bias in the sample due to nonparticipation. Further measures, such as personal contacting, multilingual instruments and interviewers, and extensive public relations, have to be taken into account. All of this can be done within smartphone-based apps. For the target groups of migrants in epidemiological studies, no singe simple solution exists [123,129,130]. However, studies indicate that constant communication and follow-up of study participants enhances the response rates in longitudinal studies and improves the quality of data [131]. For that reason, mobile apps may provide solutions for overcoming common challenges in epidemiological data collection.

### Limitations

Overall, the results of this scoping review need to be interpreted with caution. We were only able to provide a broad overview of challenges and potential benefits linked with data collection using mobile apps described in the literature. The scoping review was not focused on a specific topic in epidemiological research, in order to capture all the ideas and experiences previously described in the literature. Due to the comparatively broad (but not concrete) search algorithm that included terms related to mobile apps in combination with study designs, we might have missed some studies which have applied mobile technologies in epidemiological data collection. For example, mobile phone surveys have been excluded. However, although this scoping review was conducted in only one literature database (PubMed), we anticipate that it will provide a comprehensive overview of the potential benefits and challenges that researchers may face when using mobile apps for data collection in epidemiological studies. We were not able to identify studies specifically dealing with the target group of migrants or refugees.

### Conclusions

It appears that the challenges connected to data collection among migrant subgroups can be overcome by using the innovative technologies of mobile apps. These approaches allow the recruitment of study participants from diverse ethnic backgrounds when designed in a migration-sensitive, cross-language, and cross-cultural fashion. Continuing contact with study participants can be ensured during longitudinal studies. This is of particular relevance for mobile populations, such as refugees, who might not have reached their place of destination at the time of recruitment. Furthermore, due to the cost-effectiveness of app-based data collection, the challenges of small sample sizes and low response rates can be at least partially overcome. However, feasibility studies need to be conducted in the near future to test the applicability and acceptance of mobile apps for epidemiological research among various subpopulations.

## Appendix

Multimedia Appendix 1 Supplementary appendix.

## References

[1] Statista (2021). Anzahl der Smartphone-Nutzer weltweit von 2016 bis 2019 und Prognose bis 2023.

[2] Research 2 Guidance (2017). mHealth App Developer Economics 2015: The current status and trends of the mHealth app market.

[3] Kim JH, Kwon S, Shim SR, Sun HY, Ko YM, Chun D, Yang WJ, Song YS (2014). Validation and reliability of a smartphone application for the International Prostate Symptom Score questionnaire: a randomized repeated measures crossover study. J Med Internet Res.

[4] Ozdalga E, Ozdalga A, Ahuja N (2012). The smartphone in medicine: a review of current and potential use among physicians and students. J Med Internet Res.

[5] Behar J, Roebuck A, Domingos JS, Gederi E, Clifford GD (2013). A review of current sleep screening applications for smartphones. Physiol Meas.

[6] Pfaeffli L, Maddison R, Jiang Y, Dalleck L, Löf M (2013). Measuring Physical Activity in a Cardiac Rehabilitation Population Using a Smartphone-Based Questionnaire. J Med Internet Res.

[7] Palmier-Claus JE, Ainsworth J, Machin M, Barrowclough C, Dunn G, Barkus E, Rogers A, Wykes T, Kapur S, Buchan I, Salter E, Lewis SW (2012). The feasibility and validity of ambulatory self-report of psychotic symptoms using a smartphone software application. BMC Psychiatry.

[8] Dufau S, Duñabeitia JA, Moret-Tatay C, McGonigal A, Peeters D, Alario F, Balota DA, Brysbaert M, Carreiras M, Ferrand L, Ktori M, Perea M, Rastle K, Sasburg O, Yap MJ, Ziegler JC, Grainger J (2011). Smart phone, smart science: how the use of smartphones can revolutionize research in cognitive science. PLoS One.

[9] Abelson JS, Symer M, Peters A, Charlson M, Yeo H (2017). Mobile health apps and recovery after surgery: What are patients willing to do?. The American Journal of Surgery.

[10] BinDhim NF, Shaman AM, Trevena L, Basyouni MH, Pont LG, Alhawassi TM (2014). Depression screening via a smartphone app: cross-country user characteristics and feasibility. J Am Med Inform Assoc.

[11] Burnay E, Cruz-Correia R, Jacinto T, Sousa AS, Fonseca J (2013). Challenges of a mobile application for asthma and allergic rhinitis patient enablement-interface and synchronization. Telemed J E Health.

[12] Hartin P, Nugent C, McClean S (2014). A smartphone application to evaluate technology adoption and usage in persons with dementia.

[13] Min YH, Lee JW, Shin Y, Jo M, Sohn G, Lee J, Lee G, Jung KH, Sung J, Ko BS, Yu J, Kim HJ, Son BH, Ahn SH (2014). Daily collection of self-reporting sleep disturbance data via a smartphone app in breast cancer patients receiving chemotherapy: a feasibility study. J Med Internet Res.

[14] Mulhern B, O’Gorman H, Rotherham N, Brazier J (2015). Comparing the measurement equivalence of EQ-5D-5L across different modes of administration. Health Qual Life Outcomes.

[15] Serrano KJ, Yu M, Riley WT, Patel V, Hughes P, Marchesini K, Atienza AA (2016). Willingness to Exchange Health Information via Mobile Devices: Findings From a Population-Based Survey. Ann Fam Med.

[16] Wang J, Yao NA, Liu Y, Geng Z, Wang Y, Shen N, Zhang X, Shen M, Yuan C (2017). Development of a Smartphone Application to Monitor Pediatric Patient-Reported Outcomes. Stud Health Technol Inform.

[17] Marcano Belisario JS, Doherty K, O'Donoghue J, Ramchandani P, Majeed A, Doherty G, Morrison C, Car J (2017). A bespoke mobile application for the longitudinal assessment of depression and mood during pregnancy: protocol of a feasibility study. BMJ Open.

[18] Abu-Ghanem S, Handzel O, Ness L, Ben-Artzi-Blima M, Fait-Ghelbendorf K, Himmelfarb M (2016). Smartphone-based audiometric test for screening hearing loss in the elderly. Eur Arch Otorhinolaryngol.

[19] Brooks GC, Vittinghoff E, Iyer S, Tandon D, Kuhar P, Madsen KA, Marcus GM, Pletcher MJ, Olgin JE (2015). Accuracy and Usability of a Self-Administered 6-Minute Walk Test Smartphone Application. Circ Heart Fail.

[20] Dempster NJ, Risk R, Clark R, Meddings RN (2014). Urologists' usage and perceptions of urological apps. J Telemed Telecare.

[21] Sheehan B, Lee Y, Rodriguez M, Tiase V, Schnall R (2012). A comparison of usability factors of four mobile devices for accessing healthcare information by adolescents. Appl Clin Inform.

[22] Snipes SA, Smyth JM, Murphy D, Miranda PY, Ishino FAM (2015). Provision Increases Reported PPE Use for Mexican Immigrant Farmworkers. Journal of Occupational and Environmental Medicine.

[23] Tawara S, Yonemochi Y, Kosaka T, Kouzaki Y, Takita T, Tsuruta T (2013). Use of Patients' Mobile Phones to Store and Share Personal Health Information: Results of a Questionnaire Survey. Intern Med.

[24] Zhang MW, Ho CS, Fang P, Lu Y, Ho RC (2014). Methodology of developing a smartphone application for crisis research and its clinical application. Technol Health Care.

[25] Bajaj JS, Thacker LR, Heuman DM, Fuchs M, Sterling RK, Sanyal AJ, Puri P, Siddiqui MS, Stravitz RT, Bouneva I, Luketic V, Noble N, White MB, Monteith P, Unser A, Wade JB (2013). The Stroop smartphone application is a short and valid method to screen for minimal hepatic encephalopathy. Hepatology.

[26] Mehta S, Barker K, Bowman B, Galloway H, Oliashirazi N, Oliashirazi A (2016). Reliability, Concurrent Validity, and Minimal Detectable Change for iPhone Goniometer App in Assessing Knee Range of Motion. J Knee Surg.

[27] Arsand E, Tatara N, ostengen G, Hartvigsen G (2010). Mobile Phone-Based Self-Management Tools for Type 2 Diabetes: The Few Touch Application. Journal of Diabetes Science and Technology.

[28] Azevedo R, Bernardes M, Fonseca J, Lima A (2015). Smartphone application for rheumatoid arthritis self-management: cross-sectional study revealed the usefulness, willingness to use and patients' needs. Rheumatol Int.

[29] Becker S, Brandl C, Meister S, Nagel E, Miron-Shatz T, Mitchell A, Kribben A, Albrecht U, Mertens A (2015). Demographic and health related data of users of a mobile application to support drug adherence is associated with usage duration and intensity. PLoS One.

[30] Becker S, Kribben A, Meister S, Diamantidis CJ, Unger N, Mitchell A (2013). User Profiles of a Smartphone Application to Support Drug Adherence — Experiences from the iNephro Project. PLoS ONE.

[31] Liu X, Wang R, Zhou D, Hong Z (2016). Feasibility and acceptability of smartphone applications for seizure self-management in China: Questionnaire study among people with epilepsy. Epilepsy Behav.

[32] Reynoldson C, Stones C, Allsop M, Gardner P, Bennett MI, Closs SJ, Jones R, Knapp P (2014). Assessing the quality and usability of smartphone apps for pain self-management. Pain Med.

[33] Shah N, Jonassaint J, De Castro L (2014). Patients Welcome the Sickle Cell Disease Mobile Application to Record Symptoms Technology (SMART). Hemoglobin.

[34] Tregarthen JP, Lock J, Darcy AM (2015). Development of a smartphone application for eating disorder self-monitoring. Int J Eat Disord.

[35] Zhang MW, Ho RC, Hawa R, Sockalingam S (2015). Pilot implementation and user preferences of a Bariatric After-care application. Technol Health Care.

[36] Atkinson KM, Ducharme R, Westeinde J, Wilson SE, Deeks SL, Pascali D, Wilson K (2015). Vaccination attitudes and mobile readiness: A survey of expectant and new mothers. Human Vaccines & Immunotherapeutics.

[37] Conway N, Campbell I, Forbes P, Cunningham S, Wake D (2016). mHealth applications for diabetes: User preference and implications for app development. Health Informatics J.

[38] Alenazi H, Alghamdi M, Alradhi S, Househ M, Zakaria N (2017). A Study on Saudi Diabetic Patients' Readiness to Use Mobile Health. Stud Health Technol Inform.

[39] Swendeman D, Ramanathan N, Baetscher L, Medich M, Scheffler A, Comulada WS, Estrin D (2015). Smartphone Self-Monitoring to Support Self-Management Among People Living With HIV. J Acquir Immune Defic Syndr.

[40] Barrio P, Ortega L, López H, Gual A (2017). Self-management and Shared Decision-Making in Alcohol Dependence via a Mobile App: a Pilot Study. Int J Behav Med.

[41] Con D, Jackson B, Gray K, De Cruz P (2017). eHealth for inflammatory bowel disease self-management – the patient perspective. Scand J Gastroenterol.

[42] Crosby LE, Ware RE, Goldstein A, Walton A, Joffe NE, Vogel C, Britto MT (2017). Development and evaluation of iManage: A self-management app co-designed by adolescents with sickle cell disease. Pediatr Blood Cancer.

[43] Eisenhauer CM, Hageman PA, Rowland S, Becker BJ, Barnason SA, Pullen CH (2017). Acceptability of mHealth Technology for Self-Monitoring Eating and Activity among Rural Men. Public Health Nurs.

[44] Lee J, Song S, Ahn J, Kim Y, Lee J (2017). Use of a Mobile Application for Self-Monitoring Dietary Intake: Feasibility Test and an Intervention Study. Nutrients.

[45] Simpson AJ, Honkoop PJ, Kennington E, Snoeck-Stroband J, Smith I, East J, Coleman C, Caress A, Chung KF, Sont JK, Usmani O, Fowler S (2017). Perspectives of patients and healthcare professionals on mHealth for asthma self-management. Eur Respir J.

[46] Sommer J, Daus M, Smith M, Luna D (2017). Mobile Application for Pregnant Women: What Do Mothers Say?. Stud Health Technol Inform.

[47] Yu DX, Parmanto B, Dicianno BE, Watzlaf VJ, Seelman KD (2017). Accessibility needs and challenges of a mHealth system for patients with dexterity impairments. Disabil Rehabi Assist Technol.

[48] Jiam NT, Hoon AH, Hostetter CF, Khare MM (2017). IIAM (important information about me): a patient portability profile app for adults, children and families with neurodevelopmental disabilities. Disabil Rehabil Assist Technol.

[49] Bielli E, Carminati F, La CS, Lina M, Brunelli C, Tamburini M (2004). A Wireless Health Outcomes Monitoring System (WHOMS): development and field testing with cancer patients using mobile phones. BMC Med Inform Decis Mak.

[50] Dunton GF, Liao Y, Intille SS, Spruijt-Metz D, Pentz M (2011). Investigating children's physical activity and sedentary behavior using ecological momentary assessment with mobile phones. Obesity (Silver Spring).

[51] Eagle N, Pentland A, Lazer D (2009). Inferring friendship network structure by using mobile phone data. Proceedings of the National Academy of Sciences.

[52] Jovanov E, Frith K, Anderson F, Milosevic M, Shrove M (2011). Real-time monitoring of occupational stress of nurses.

[53] Dewulf B, Neutens T, Lefebvre W, Seynaeve G, Vanpoucke C, Beckx C, Van de Weghe N (2016). Dynamic assessment of exposure to air pollution using mobile phone data. Int J Health Geogr.

[54] Saeb Sohrab, Zhang Mi, Karr Christopher J, Schueller Stephen M, Corden Marya E, Kording Konrad P, Mohr David C (2015). Mobile Phone Sensor Correlates of Depressive Symptom Severity in Daily-Life Behavior: An Exploratory Study. J Med Internet Res.

[55] McLean A, Osgood N, Newstead-Angel J, Stanley K, Knowles D, van der Kamp W, Qian W, Dyck R (2017). Building Research Capacity: Results of a Feasibility Study Using a Novel mHealth Epidemiological Data Collection System Within a Gestational Diabetes Population. Stud Health Technol Inform.

[56] Katib A, Rao D, Rao P, Williams K, Grant J (2015). A prototype of a novel cell phone application for tracking the vaccination coverage of children in rural communities. Comput Methods Programs Biomed.

[57] Haskew J, Kenyi V, William J, Alum R, Puri A, Mostafa Y, Davis R (2015). Use of Mobile Information Technology during Planning, Implementation and Evaluation of a Polio Campaign in South Sudan. PLoS ONE.

[58] Rajput ZA, Mbugua S, Amadi D, Chepngeno V, Saleem JJ, Anokwa Y, Hartung C, Borriello G, Mamlin BW, Ndege SK, Were MC (2012). Evaluation of an Android-based mHealth system for population surveillance in developing countries. J Am Med Inform Assoc.

[59] Rotheram-Borus M, Richter L, Van RH, van HA, Tomlinson M, Stein A, Rochat T, de KJ, Mtungwa N, Mkhize L, Ndlovu L, Ntombela L, Comulada WS, Desmond KA, Greco E (2011). Project Masihambisane: a cluster randomised controlled trial with peer mentors to improve outcomes for pregnant mothers living with HIV. Trials.

[60] Setswe G, Muyanga S, Witthun J, Nyasulu P (2015). Public awareness and knowledge of the National Health Insurance in South Africa. Pan Afr Med J.

[61] Tomlinson M, Solomon W, Singh Y, Doherty T, Chopra M, Ijumba P, Tsai AC, Jackson D (2009). The use of mobile phones as a data collection tool: a report from a household survey in South Africa. BMC Med Inform Decis Mak.

[62] Vélez O, Okyere PB, Kanter AS, Bakken S (2014). A usability study of a mobile health application for rural Ghanaian midwives. J Midwifery Womens Health.

[63] Zhang S, Wu Q, van Velthoven MH, Chen L, Car J, Rudan I, Zhang Y, Li Y, Scherpbier RW (2012). Smartphone Versus Pen-and-Paper Data Collection of Infant Feeding Practices in Rural China. J Med Internet Res.

[64] Knoble SJ, Bhusal MR (2015). Electronic diagnostic algorithms to assist mid-level health care workers in Nepal: A mixed-method exploratory study. Int J Med Inform.

[65] Andrew BY, Stack CM, Yang JP, Dodds JA (2017). mStroke: “Mobile Stroke”—Improving Acute Stroke Care with Smartphone Technology. J Stroke Cerebrovasc Dis.

[66] Bakibinga P, Kamande E, Omuya M, Ziraba AK, Kyobutungi C (2017). The role of a decision-support smartphone application in enhancing community health volunteers' effectiveness to improve maternal and newborn outcomes in Nairobi, Kenya: quasi-experimental research protocol. BMJ Open.

[67] Coppock D, Zambo D, Moyo D, Tanthuma G, Chapman J, Re VL, Graziani A, Lowenthal E, Hanrahan N, Littman-Quinn R, Kovarik C, Albarracin D, Holmes JH, Gross R (2018). Development and Usability of a Smartphone Application for Tracking Antiretroviral Medication Refill Data for Human Immunodeficiency Virus. Methods Inf Med.

[68] Kenny A, Gordon N, Griffiths T, Kraemer JD, Siedner MJ (2017). Validation Relaxation: A Quality Assurance Strategy for Electronic Data Collection. J Med Internet Res.

[69] Mishori R, Anastario M, Naimer K, Varanasi S, Ferdowsian H, Abel D, Chugh K (2017). mJustice: Preliminary Development of a Mobile App for Medical-Forensic Documentation of Sexual Violence in Low-Resource Environments and Conflict Zones. Glob Health Sci Pract.

[70] Modi D, Desai S, Dave K, Shah S, Desai G, Dholakia N, Gopalan R, Shah P (2017). Cluster randomized trial of a mHealth intervention “ImTeCHO” to improve delivery of proven maternal, neonatal, and child care interventions through community-based Accredited Social Health Activists (ASHAs) by enhancing their motivation and strengthening supervision in tribal areas of Gujarat, India: study protocol for a randomized controlled trial. Trials.

[71] Motulsky A, Wong J, Cordeau J, Pomalaza J, Barkun J, Tamblyn R (2016). Using mobile devices for inpatient rounding and handoffs: an innovative application developed and rapidly adopted by clinicians in a pediatric hospital. J Am Med Inform Assoc.

[72] Vedachalam S, MacDonald LH, Shiferaw S, Seme A, Schwab KJ (2017). Underreporting of high-risk water and sanitation practices undermines progress on global targets. PLoS ONE.

[73] Zhou L, Watzlaf V, Abernathy P, Abdelhak M (2017). A Health Information System for Scalable and Comprehensive Assessment of Well-Being: A Multidisciplinary Team Solution. Perspect Health Inf Manag.

[74] Runyan JD, Steenbergh TA, Bainbridge C, Daugherty DA, Oke L, Fry BN (2013). A smartphone ecological momentary assessment/intervention. PLoS One.

[75] Spook JE, Paulussen T, Kok G, Van EP (2013). Monitoring dietary intake and physical activity electronically: feasibility, usability, and ecological validity of a mobile-based Ecological Momentary Assessment tool. J Med Internet Res.

[76] Webb JR, Webb BF, Schroeder MC, North CS (2013). Association of aphthous ulcers with self-reported symptoms of depression in a sample of smartphone users. Ann Clin Psychiatry.

[77] Seto E, Hua J, Wu L, Shia V, Eom S, Wang M, Li Y (2016). Models of Individual Dietary Behavior Based on Smartphone Data: The Influence of Routine, Physical Activity, Emotion, and Food Environment. PLoS One.

[78] Reid SC, Kauer SD, Dudgeon P, Sanci LA, Shrier LA, Patton GC (2009). A mobile phone program to track young people's experiences of mood, stress and coping. Development and testing of the mobiletype program. Soc Psychiatry Psychiatr Epidemiol.

[79] Stinson JN, Jibb LA, Nguyen C, Nathan PC, Maloney AM, Dupuis LL, Gerstle JT, Hopyan S, Alman BA, Strahlendorf C, Portwine C, Johnston DL (2015). Construct validity and reliability of a real-time multidimensional smartphone app to assess pain in children and adolescents with cancer. Pain.

[80] Brannon EE, Cushing CC, Crick CJ, Mitchell TB (2016). The promise of wearable sensors and ecological momentary assessment measures for dynamical systems modeling in adolescents: a feasibility and acceptability study. Transl Behav Med.

[81] Warren-Stomberg M, Jacobsson J, Brattwall M, Jildenstål P (2016). At-home monitoring after surgery/anaesthesia - a challenge. J Eval Clin Pract.

[82] Alqahtani AS, Rashid H, Basyouni MH, Alhawassi TM, BinDhim NF (2017). Public response to MERS-CoV in the Middle East: iPhone survey in six countries. J Infect Public Health.

[83] Chen Y, Wong J, Ayob A, Othman N, Poh B (2017). Can Malaysian Young Adults Report Dietary Intake Using a Food Diary Mobile Application? A Pilot Study on Acceptability and Compliance. Nutrients.

[84] Farnham A, Furrer R, Blanke U, Stone E, Hatz C, Puhan M (2017). The quantified self during travel: mapping health in a prospective cohort of travellers. J Travel Med.

[85] Fernández-Castro J, Martínez-Zaragoza F, Rovira T, Edo S, Solanes-Puchol ?, Martín-del-Río B, García-Sierra R, Benavides-Gil G, Doval E (2017). How does emotional exhaustion influence work stress? Relationships between stressor appraisals, hedonic tone, and fatigue in nurses’ daily tasks: A longitudinal cohort study. Int J Nurs Stud.

[86] Gomes MS, Bonan PRF, Ferreira VYN, de Lucena Pereira L, Correia RJC, da Silva Teixeira HB, Pereira DC, Bonan P (2017). Development of a mobile application for oral cancer screening. Technol Health Care.

[87] Hennig T, Krkovic K, Lincoln TM (2017). What predicts inattention in adolescents? An experience-sampling study comparing chronotype, subjective, and objective sleep parameters. Sleep Med.

[88] Kaplan-Neeman R, Muchnik C, Amir N (2017). Listening to music with personal listening devices: monitoring the noise dose using a smartphone application. Int J Audiol.

[89] Liddle J, Wishink A, Springfield L, Gustafsson L, Ireland D, Silburn P (2017). Can smartphones measure momentary quality of life and participation? A proof of concept using experience sampling surveys with university students. Aust Occup Ther J.

[90] Mindell JA, Leichman ES, Walters RM (2017). Sleep location and parent-perceived sleep outcomes in older infants. Sleep Med.

[91] Moore RC, Kaufmann CN, Rooney AS, Moore DJ, Eyler LT, Granholm E, Woods SP, Swendsen J, Heaton RK, Scott J, Depp CA (2017). Feasibility and Acceptability of Ecological Momentary Assessment of Daily Functioning Among Older Adults with HIV. Am J Geriatr Psychiatry.

[92] Moran EK, Culbreth AJ, Barch DM (2017). Ecological momentary assessment of negative symptoms in schizophrenia: Relationships to effort-based decision making and reinforcement learning. J Abnorm Psychol.

[93] Roberts ME, Lu B, Browning CR, Ferketich AK (2017). Tracking Young Adults' Attitudes Toward Tobacco Marketing Using Ecological Momentary Assessment (EMA). Subst Use Misuse.

[94] Roy R, Rangan A, Hebden L, Yu Louie JC, Tang LM, Kay J, Allman-Farinelli M (2017). Dietary contribution of foods and beverages sold within a university campus and its effect on diet quality of young adults. Nutrition.

[95] Sperry SH, Kwapil TR (2017). What can daily life assessment tell us about the bipolar spectrum?. Psychiatry Res.

[96] Timmer BHB, Hickson L, Launer S (2017). Ecological Momentary Assessment: Feasibility, Construct Validity, and Future Applications. Am J Audiol.

[97] van Wel L, Huss A, Bachmann P, Zahner M, Kromhout H, Fröhlich J, Vermeulen R (2017). Context-sensitive ecological momentary assessments; integrating real-time exposure measurements, data-analytics and health assessment using a smartphone application. Environ Int.

[98] Wang J, Wang Q, Wimalaratne I, Menkes DB, Wang X (2017). Chinese non-psychiatric hospital doctors’ attitudes toward management of psychological/psychiatric problems. BMC Health Serv Res.

[99] Worthen-Chaudhari L, McGonigal J, Logan K, Bockbrader MA, Yeates KO, Mysiw WJ (2017). Reducing concussion symptoms among teenage youth: Evaluation of a mobile health app. Brain Inj.

[100] Pelletier J, Rowe M, François N, Bordeleau J, Lupien S (2013). No personalization without participation: on the active contribution of psychiatric patients to the development of a mobile application for mental health. BMC Med Inform Decis Mak.

[101] Bousquet J, Bewick M, Arnavielhe S, Mathieu-Dupas E, Murray R, Bedbrook A, Caimmi DP, Vandenplas O, Hellings PW, Bachert C, Anto JM, Bergmann KC, Bindslev-Jensen C, Bosnic-Anticevich S, Bouchard J, Canonica GW, Chavannes NH, Cruz AA, Dahl R, Demoly P, De Vries G, Devillier P, Fink-Wagner A, Fokkens WJ, Fonseca J, Guldemond NA, Haahtela T, Hellqvist-Dahl B, Just J, Keil T, Klimek L, Kowalski ML, Kuna P, Kvedariene V, Laune D, Larenas-Linnemann D, Mullol J, Pereira AM, Carreiro-Martins P, Melén E, Morais-Almeida M, Nogueira-Silva L, O'Hehir RE, Papadopoulos NG, Passalacqua G, Portejoie F, Price D, Ryan D, Samolinski B, Sheikh A, Simons FER, Spranger O, Todo Bom A, Tomazic PV, Triggiani M, Valero A, Valovirta E, Valiulis A, van Eerd M, Wickman M, Young I, Zuberbier T (2017). Work productivity in rhinitis using cell phones: The MASK pilot study. Allergy.

[102] Marcano Belisario JS, Jamsek J, Huckvale K, O'Donoghue J, Morrison CP, Car J (2015). Comparison of self-administered survey questionnaire responses collected using mobile apps versus other methods. Cochrane Database Syst Rev.

[103] Pombo N, Garcia N, Bousson K, Spinsante S, Chorbev I (2016). Pain Assessment–Can it be Done with a Computerised System? A Systematic Review and Meta-Analysis. Int J Environ Res Pub Health.

[104] Udtha M, Nomie K, Yu E, Sanner J (2014). Novel and Emerging Strategies for Longitudinal Data Collection. J Nurs Scholarsh.

[105] Gibson DG, Pereira A, Farrenkopf BA, Labrique AB, Pariyo GW, Hyder AA (2017). Mobile Phone Surveys for Collecting Population-Level Estimates in Low- and Middle-Income Countries: A Literature Review. J Med Internet Res.

[106] Bot BM, Suver C, Neto EC, Kellen M, Klein A, Bare C, Doerr M, Pratap A, Wilbanks J, Dorsey ER, Friend SH, Trister AD (2016). The mPower study, Parkinson disease mobile data collected using ResearchKit. Sci Data.

[107] Chan YY, Wang P, Rogers L, Tignor N, Zweig M, Hershman SG, Genes N, Scott ER, Krock E, Badgeley M, Edgar R, Violante S, Wright R, Powell CA, Dudley JT, Schadt EE (2017). The Asthma Mobile Health Study, a large-scale clinical observational study using ResearchKit. Nat Biotechnol.

[108] Demirci JR, Bogen DL (2017). An Ecological Momentary Assessment of Primiparous Women’s Breastfeeding Behavior and Problems From Birth to 8 Weeks. J Hum Lact.

[109] Faherty LJ, Hantsoo L, Appleby D, Sammel MD, Bennett IM, Wiebe DJ (2017). Movement patterns in women at risk for perinatal depression: use of a mood-monitoring mobile application in pregnancy. J Am Med Inform Assoc.

[110] Horsch CH, Lancee J, Griffioen-Both F, Spruit S, Fitrianie S, Neerincx MA, Beun RJ, Brinkman W (2017). Mobile Phone-Delivered Cognitive Behavioral Therapy for Insomnia: A Randomized Waitlist Controlled Trial. J Med Internet Res.

[111] Isetta V, Torres M, González K, Ruiz C, Dalmases M, Embid C, Navajas D, Farré R, Montserrat JM (2017). A New mHealth application to support treatment of sleep apnoea patients. J Telemed Telecare.

[112] Jamison RN, Jurcik DC, Edwards RR, Huang C, Ross EL (2017). A Pilot Comparison of a Smartphone App With or Without 2-Way Messaging Among Chronic Pain Patients: Who Benefits From a Pain App?. Clin J Pain.

[113] Noë B, Turner LD, Linden DEJ, Allen SM, Maio GR, Whitaker RM (2017). Timing rather than user traits mediates mood sampling on smartphones. BMC Res Notes.

[114] Silva de Lima AL, Hahn T, Evers LJW, de Vries NM, Cohen E, Afek M, Bataille L, Daeschler M, Claes K, Boroojerdi B, Terricabras D, Little MA, Baldus H, Bloem BR, Faber MJ (2017). Feasibility of large-scale deployment of multiple wearable sensors in Parkinson's disease. PLoS ONE.

[115] Sundberg K, Wengström Y, Blomberg K, Hälleberg-Nyman M, Frank C, Langius-Eklöf A (2017). Early detection and management of symptoms using an interactive smartphone application (Interaktor) during radiotherapy for prostate cancer. Support Care Cancer.

[116] Cooray C, Matusevicius M, Wahlgren N, Ahmed N (2015). Mobile Phone–Based Questionnaire for Assessing 3 Months Modified Rankin Score After Acute Stroke. Circ Cardiovasc Qual Outcomes.

[117] Pavliscsak H, Little JR, Poropatich RK, McVeigh FL, Tong J, Tillman JS, Smith CH, Fonda SJ (2015). Assessment of patient engagement with a mobile application among service members in transition. J Am Med Inform Assoc.

[118] Symer MM, Abelson JS, Milsom J, McClure B, Yeo HL (2017). A Mobile Health Application to Track Patients After Gastrointestinal Surgery: Results from a Pilot Study. J Gastrointest Surg.

[119] Labhart F, Anderson KG, Kuntsche E (2017). The Spirit Is Willing, But the Flesh is Weak: Why Young People Drink More Than Intended on Weekend Nights-An Event-Level Study. Alcohol Clin Exp Res.

[120] Olson D, Lamb M, Lopez MR, Colborn K, Paniagua-Avila A, Zacarias A, Zambrano-Perilla R, Rodríguez-Castro SR, Cordon-Rosales C, Asturias EJ (2017). Performance of a Mobile Phone App-Based Participatory Syndromic Surveillance System for Acute Febrile Illness and Acute Gastroenteritis in Rural Guatemala. J Med Internet Res.

[121] Burke LE, Shiffman S, Music E, Styn MA, Kriska A, Smailagic A, Siewiorek D, Ewing LJ, Chasens E, French B, Mancino J, Mendez D, Strollo P, Rathbun SL (2017). Ecological Momentary Assessment in Behavioral Research: Addressing Technological and Human Participant Challenges. J Med Internet Res.

[122] Peleg M, Shahar Y, Quaglini S, Broens T, Budasu R, Fung N, Fux A, García-Sáez G, Goldstein A, González-Ferrer A, Hermens H, Hernando ME, Jones V, Klebanov G, Klimov D, Knoppel D, Larburu N, Marcos C, Martínez-Sarriegui I, Napolitano C, Pallàs �, Palomares A, Parimbelli E, Pons B, Rigla M, Sacchi L, Shalom E, Soffer P, van SB (2017). Assessment of a personalized and distributed patient guidance system. Int J Med Inform.

[123] Lepkowski J (1991). Sampling the difficult-to-sample. J Nutr.

[124] Rechel B, Mladovsky P, Devillé W (2012). Monitoring migrant health in Europe: A narrative review of data collection practices. Health Policy.

[125] (2018). General Data Protection Regulation.

[126] WHO (2010). Health of migrants: The way forward, report of a global consultation, Madrid, Spain, 3 - 5 March.

[127] Ingleby D (2009). European research on migration and health: Background paper for the AMAC project.

[128] Zeeb H, Razum O (2006). Epidemiologische Studien in der Migrationsforschung. Bundesgesundheitsbl.

[129] Reiss K, Makarova N, Spallek J, Zeeb H, Razum O (2012). Identifizierung und Rekrutierung von Menschen mit Migrationshintergrund für epidemiologische Studien in Deutschland. Gesundheitswesen.

[130] Reiss K, Dragano N, Ellert U, Fricke J, Greiser KH, Keil T, Krist L, Moebus S, Pundt N, Schlaud M, Yesil-Jurgens R, Zeeb H, Zimmermann H, Razum O, Jockel K, Becher H (2014). Comparing sampling strategies to recruit migrants for an epidemiological study. Results from a German feasibility study. Eur J Public Health.

[131] Garduño-Diaz SD, Husain W, Ashkanani F, Khokhar S (2013). Meeting challenges related to the dietary assessment of ethnic minority populations. J Hum Nutr Diet.

