# Supplementary material for: Possibilities, Problems, and Perspectives of Data Collection by Mobile Apps in Longitudinal Epidemiological Studies: Scoping Review
Source: J Med Internet Res. 2021 Jan 22;23(1):e17691. doi: 10.2196/17691 (PMC7864774; doi:10.2196/17691)
Supplement: Multimedia Appendix 1 [file jmir_v23i1e17691_app1.pdf]

Supplementary Appendix: Details of studies included in the scoping review

Reference	Publication type	Country	Study design	Indication area	Area of interest (instruments)	Objective	Duration		Number of participants	Age (groups)
							Recruitment	Survey period		
Bot et al. (2016)	Feasibility study/ (first) study results	USA	Clinical observational	Parkinson disease (patients and controls)	Changes in symptoms of Parkinson disease over time (PDQ-8, subset from MDS-UPDRS plus 4 tasks)	Should help to establish baseline variability of real-world activity measurement collected via mobile phone	Still running	(First) 6 months (app and study is still running)	N=8,320 (completed at least 1 survey/task) N=898 (completion more than 1 time)	--
Burke et al. (2017)	Pilot/ feasibility study	Pittsburgh, USA	EMA assessment over one years	Weight loss and eating behavior in obese people	Examine the triggers of lapses and relapse following intentional weight loss Lessons learned from the EMA data collection process	Provide a practical guide to developing and implementing a longitudinal EMA study	Enrollment completed in January 2014 after 2 years of enrollment in 6 cohorts	12 months (study completion in March 2015 with 87.4% retention rate)	N=151	Average age: 51 (±10) years
Chan et al. (2017)	Initial findings (from the Asthma Mobile Health Study)	Across USA	Clinical observational (large-scale)	Asthma patients	Examine health status and healthcare uptake of user: Asthma symptoms, presumed triggers, medication adherence as well as Participants' healthcare utilization (HCU), quality of life (EQ-5D)	(i) Feasibility of smartphone-based recruitment; (ii) characteristics of a study cohort recruited through the ResearchKit platform; (iii) user engagement and retention patterns; and (iv) user data sharing preferences	From app relaunch 6 months onwards: participants had to fill out a series of intake surveys over four consecutive days after enrollment	6 months	N=2,317 'robust users' (R) (included participants who completed at least five daily or weeks surveys)<br><br>(N=7,593 'baseline users' (B), respond at least once)	18-34: 54% B, 52% R<br><br>35-64: 41% B, 44% R<br><br>65+: 0.05% B,<br>0.04% R
Cooray et al. (2015)	Study results	Stockholm, Sweden	Cohort	Acute stroke patients	Find an alternative in stroke follow up via mRS	Assess modified ranking score (mRS) as a mobile phone questionnaire instead of clinical assessment	Two months (March to May 2014)	--	N=48	Mean age:67
Demirci and Bogen (2017)	Study results	USA	Prospective observational	Primiparous women<br><br>High income	Describe early, 'real time' breastfeeding behaviors and perceived problems of primiparous women	(i) Early, unintended breastfeeding reduction and cessation	October 2014 to August 2015 during postpartum hospitalization	8 weeks	N=61, 48 of whom provided app data and/or completed	18-35 years

Reference	Publication type	Country	Study design	Indication area	Area of interest (instruments)	Objective	Duration		Number of participants	Age (groups)
							Recruitment	Survey period		
					(Iowa Infant Feeding Attitude Scale, PROMIS Emotional Distress-Anxiety Scale SF, Perceived Stress Scale)	(ii) inform timing and content of another technology-based intervention to deliver targeted breastfeeding support			FU interviews	
Faherty et al. (2017)	Study results	USA	Cohort	Pregnant women at risk for perinatal depression<br><br>Low income	Examine if mood (PHQ-9, GAD-7) is associated with daily movement patterns	Examine the association of self-reported daily mood and passively collected data with movement patterns of pregnant women at risk for perinatal depression	--	8 weeks	N=36	Mean age: 25.7 years
Horsch et al. (2017)	Study results	Netherlands	RCT	Participants with relatively mild insomnia disorder<br>High percentage of university-educated students	Investigating cognitive behavioral therapy for insomnia (CBT-I) delivered by a fully automated mobile phone app	Examine the efficacy of CBT-I delivered via the Sleepcare mobile phone app compared with a waitlist control group	August to October 2015 via websites, social media, online advertisements, flyers, and press release	6 to 7 weeks program (depends on participant's adherence) duration plus 3-months FU	N=151 (74 app use, 79 waitlist)	Mean age: 39.7 (SD: 13.4, range 18-80) years
Isetta et al. (2017)	Pilot study	Barcelona, Spain	Observational	Patients with Obstructive sleep apnea (OSA)<br>mostly male, middle-aged, and overweight	Promoting self-monitoring of Continuous positive airway pressure (CPAP) (Epworth sleepiness scale)	Assess the feasibility and acceptability of APPnea	--	6 weeks of app use (72%±24% completed the daily questionnaire for an average of the six weeks, 63% of them were regular users)	N=60	Mean age: 56 (±10) years
Jamison et al. (2017)	Pilot study	Boston, USA	Observational	Chronic pain patients<br>(heavy pain and on average about 10 years)	Self-monitoring of symptoms and communicating with provider (various instruments)	Determine the effect of introducing a smartphone pain app that enables chronic pain patients to assess, monitor and communicate their status to the provider	--	3 months (plus option for 6 months)	N=105 (N=90 downloaded the app and 82 of the participants submitted daily reports)	Mean age: 47.1 years, range 18-72

Reference	Publication type	Country	Study design	Indication area	Area of interest (instruments)	Objective	Duration		Number of participants	Age (groups)
							Recruitment	Survey period		
Labhart et al. (2017)	Event-level study	Lausanne and Zurich, Switzerland	Observational	Young adults (who drink alcohol on weekend nights)	Documenting young adults' nightlife behaviors using event-level questionnaires, pictures, videos, and GPS, accelerometers, and Bluetooth	Drinking intentions, individual and situational factor associated with a subsequent consumption over the course of multiple nights	September 2014	7 weeks	N=176	Mean age: 19.1 years, range 16-25
Noe et al. (2017)	Study results	Cardiff, Wales	Observational	Mood tracking (healthy people)	Mood tracking via two different methods (current mood and daily mood at end of the day)	Investigate the reasons to favor collecting mood through current or daily mood surveys and outlines design recommendations for mood sampling using smartphones	--	8 weeks	N=76 (N=64 available smartphone data)	Mean age: 24.94 (±5.69) years range: 19 and 46
Olson et al. (2017)	Study results	Rural Guatemala	Prospective cohort	Acute febrile illness and acute gastroenteritis	To detect norovirus and dengue virus in a resource limited setting	Study the performance of a mobile phone app-based participatory syndromic surveillance system for collecting syndromic data (acute febrile illness and acute gastroenteritis)	April to September 2015	October 2015 -June 2016, weekly FU	N=469 children in 207 households	Mean age of children: 7.3 (±4.7)
Pavliscsak et al. (2016)	Feasibility study	Oxford, England	RCT	Patients with behavioral problems, PTSD and/ or TBI in rehabilitation	Test the clinical efficacy of mCare vs standard care	Examine study participants' engagement with mCare	--	36 weeks (up to enrollment)	N=95	Average age: 35 and 40
Peleg et al. (2017)	Feasibility study/ small pilot study	Italy (AF) and Spain (GDM)	Cohort	Atrial fibrillation (AF) and gestational diabetes mellitus (GDM),<br><br>GDM more educated and younger than AF	Satisfaction with the system and quality of life in AF and GDM patients (EuroQoL and AFEQT)<br><br>Monitoring symptoms, prescription and medication reminder	Assess the system's (MobiGuide) feasibility and potential effects on patients and care providers in two different clinical domains (support decision making and symptom reporting)	--	April to December 2015. average days of use: AF patients: 127.2±68.6 days, GDM patients: 57.1±21.5 days	N=10 (AF)<br>N=19 (GDM)	GDM: mean age: 35 years

Reference	Publication type	Country	Study design	Indication area	Area of interest (instruments)	Objective	Duration		Number of participants	Age (groups)
							Recruitment	Survey period		
Silva de Lima et al. (2017)	Study results	Netherlands (NL) and North America (NAM)	Observational, two-cohort	Parkinson disease (PD)	Collect information about PD patients (movement patterns, medication intake, symptom collection, depression status via smartphone sensors and self-reports	Investigate the feasibility of the technology in different contexts	--	13 weeks (NL), 6 weeks (NAM)	N=953 (NL: 304, NAM. 649)	Data contributors: ≤50: 10% NL, 21% NAM, 51-69: 72% NL, 63% NAM ≥70: 18% NL, 16% NAM
Sundberg et al. (2017)	Study results	Sweden	Non-randomized controlled interventional study	Prostate cancer with radiotherapy (RT)	Early detection, reporting and management of symptoms, and concerns during treatment (EORTC QLQ-C30 and its module PR25, Sense of Coherence questionnaire)	Evaluate the effect on symptom burden and quality of life when using the application for real-time symptom assessment and management during radiotherapy for localized prostate cancer	--	5-8 weeks during RT plus following 3 weeks after treatment	N=130 (IG: 66, CG: 64)	Mean age (of all included): 69 years (range 52-82)
Symer et al. (2017)	Pilot study	New York, USA	Observational	Adults undergoing abdominal surgery	Track patients' recovery after gastrointestinal surgery (report pain, answer surveys, photograph their wound and reminder to stay hydrated) to prevent surgical readmission	Track feasibility of mobile application use to track patient recovery<br>Improve return to baseline activity	September 2015 – January 2017 (colorectal surgery clinics)	4 weeks after discharge from hospital	N=31	Mean age: 51.7 years (range 21-75)
